# Host-Derived *Delta-Like Canonical Notch Ligand 1* as a Novel Diagnostic Biomarker for Bacterial Sepsis—Results From a Combinational Secondary Analysis

**DOI:** 10.3389/fcimb.2019.00267

**Published:** 2019-07-23

**Authors:** Dagmar Hildebrand, Sebastian O. Decker, Christian Koch, Felix C. F. Schmitt, Sophie Ruhrmann, Emmanuel Schneck, Michael Sander, Markus Alexander Weigand, Thorsten Brenner, Klaus Heeg, Florian Uhle

**Affiliations:** ^1^Medical Microbiology and Hygiene, Centre for Infectious Diseases, Heidelberg University Hospital, Heidelberg, Germany; ^2^Department of Anesthesiology, Heidelberg University Hospital, Heidelberg, Germany; ^3^Department of Anesthesiology, Intensive Care Medicine and Pain Therapy, University Hospital of Giessen and Marburg, Giessen, Germany

**Keywords:** trauma, SIRS, sepsis, monocytes, shock, inflammation, infection, DLL1

## Abstract

**Background:** Sepsis is a life-threatening syndrome, resulting from a dysbalanced host response to infection. However, especially the early, pro-inflammatory immune response in sepsis is similar to other inflammatory conditions without infectious cause, e.g., trauma or surgery. This aspect challenges the value of current biomarkers for diagnosis, as these are often broadly induced. We earlier identified *Delta-like Protein 1* (DLL1), a canonical Notch ligand, to be released from monocytes upon bacterial stimulation. Considering the importance of monocytes in the pathophysiology of sepsis, we hypothesized that this mechanism might occur also in the clinical setting and DLL1 might serve as a biomarker of life-threatening bacterial infection.

**Methods:** We combined samples from three different studies, including subgroups of patients with sepsis (*n* = 80), surgical patients (*n* = 50), trauma patients (*n* = 36), as well as healthy controls (*n* = 50). We assessed plasma concentrations of DLL1 using ELISA. We performed Area-under-receiver-operator-curve (AUROC) analysis to evaluate the diagnostic performance of DLL1 compared to leucocytes, C-reactive protein (CRP), and procalcitonin (PCT).

**Results:** Plasma concentrations of DLL1 were strongly elevated already at sepsis onset and maintained elevated until day 7. In contrast, neither surgical patients nor patients after severe trauma presented with elevated levels, while conventional biomarkers of inflammation (e.g., leucocytes and CRP), responded. AUROC analysis revealed a cut-off of 30 ng/ml associated with the best diagnostic performance, yielding a superior accuracy of 91% for DLL1, compared to 75, 79, and 81% for CRP, leucocytes, and PCT.

**Conclusion:** DLL1 is a novel host-derived biomarker for the diagnosis of sepsis with a better performance compared to established ones, most likely due to its high robustness in non-infectious inflammatory responses.

**Clinical Trial Registration:** POCSEP-Trial DRKS00008090; MIRSI DRKS00005463; SPRINT DRKS00010991.

## Introduction

Since decades, extensive research is conducted to identify biomarkers of sepsis. In 2010, a total of 178 published biomarkers were identified, and new ones are emerging nearly on a daily base (Pierrakos and Vincent, 2010). Most of the markers solely possess prognostic values and thereby lacks the actionable result a clinician is urgently expecting from a biomarker by aiding diagnosis, response prediction or therapeutic monitoring. Considering the few markers with diagnostic value, close to none have come the long way to clinical routine. In 2016, sepsis has been re-defined as life-threatening organ dysfunction, arising from the dysregulated host response to infection (Singer et al., 2016). Therefore, the purpose of a biomarker is to clarify, if the critical condition of the patient is a result of infection demanding antibiotic treatment, or—most probably of greater importance—caused by other reasons. Latter will make unnecessary exposure to antibiotics obsolete and will free resources to proceed with extended diagnostics. However, the urgent clinical need for rapid diagnosis derives from the evidence that each hour of delayed antibiotic treatment increases mortality between 2 and 4% (Bloos et al., 2017; Seymour et al., 2017).

Today, the most abundant biomarkers measured in clinical routine are C-reactive protein (CRP) and procalcitonin (PCT) (Biron et al., 2015). PCT is often claimed as “gold standard” for the diagnosis of infection (or even sepsis). However, while evidence for PCTs usefulness in the decision for the discontinuation of antibiotic treatment is steadily growing (Rhee, 2017), its diagnostic value is under steady debate and largely depends on the clinical context of assessment (Tang et al., 2007; Wacker et al., 2013). Compared to PCT, the acute phase protein CRP is often supposed to be of lower specificity, as also conditions as surgery, cancer or severe trauma can increase its plasma levels. However, this kinetic also holds true for PCT (Parli et al., 2018). This unravels a statistical misconception and dilemma: the essential indices of a diagnostic test, above all sensitivity and specificity, are a result of a dichotomous grouping of patients according to an arbitrary threshold. By adapting this, sensitivity and specificity inevitably change as well. In real life, however, adaptions of thresholds are hardly manageable, rising the need for the identification of robust biomarkers for diagnosing life-threatening infection (alias sepsis) in as diverse as possible clinical settings and patient populations.

*Delta-like canonical Notch ligand 1* (DLL1), among others, belongs to the Delta/Jagged family of transmembrane proteins (Kovall et al., 2017). As a ligand of the Notch receptors, cell-surface DLL1 activates downstream signaling pathways upon receptor binding, which are critically involved in a plethora of processes during embryonic development, angiogenesis and hematopoiesis (Bray, 2016). Contrastingly, uncontrolled activation of Notch signaling has been connected to disturbances in development and cancer (Penton et al., 2012; Capaccione and Pine, 2013), hinting toward Notch and its ligand as therapeutic targets as well (Briot and Iruela-Arispe, 2015). As a result of the interaction, receptor and its ligand DLL1 are cleaved enzymatically from the surface, resulting in the generation of soluble DLL1 (sDLL1).

We recently found an upregulation of DLL1 in primary human monocytes in response to *in vitro* infection with various bacteria (Hildebrand et al., 2018). As a consequence, DLL1 triggered Notch signaling in neighboring cells and amplified the pro-inflammatory cytokine response. Not surprisingly, also large amounts of sDLL1 were detectable in the cell supernatant. Considering the central role of monocytes and macrophages in the pathophysiology of sepsis, we asked if this might occur in the clinical setting during human sepsis as well.

To address this, we conducted a secondary analysis of plasma samples from three independent studies, combining patients with sepsis as well as patients after surgery, trauma, and healthy volunteers. We aimed to unravel the kinetic of DLL1 and its diagnostic value as a host-derived response biomarker to discriminate sepsis from sterile systemic inflammatory processes compared to established markers.

## Materials and Methods

### Study Cohorts

The secondary analysis contains samples from three prior observational cohort studies, conducted to evaluate different biomarkers in sepsis or severe trauma. All studies have received clearance from the responsible ethics committees before recruitment. If necessary, the secondary analysis of samples has been amended. In all studies, written informed consent was obtained from the patients before inclusion. If the patient was not able to give consent, its legal representative was asked instead.

Cohort 1 (POCSEP-Trial; German Clinical Trials Register ID: DRKS00008090; Ethical committee of the Medical Faculty of the University Heidelberg: S-247/2014) contains samples from 30 adult patients with sepsis or septic shock according to the 2001 consensus criteria (Levy et al., 2003), drawn on onset (0 h), 24 h, 48 h, and 7 days later. As control groups, 30 healthy volunteers, as well as 30 patients after extensive visceral surgery (e.g., Whipple procedure or hemihepatectomy) were included with samples available from 0, 24, and 48 h after end of surgery. Exclusion criteria were recent cardiac surgery, severe trauma or therapy with tranexamic acid (Schmitt et al., 2019).

Cohort 2 (MIRSI-Trial; German Clinical Trials Register ID: DRKS00005463; Ethical committee of the Medical Faculty of the University Heidelberg: S-097/2013) contains samples from 50 patients with septic shock according to the 2001 consensus criteria (Levy et al., 2003), drawn on onset (0 h), 24 h, 48 h, and 7 days later. Same control groups as described in Cohort 1 have been recruited with 20 patients and 20 healthy volunteers, respectively. No exclusion criteria were applied (Decker et al., 2017).

Despite patients with sepsis were recruited under the consensus criteria valid at time of study conduct, all patients also fulfilled Sepsis-3 consensus criteria as evaluated retrospectively. Importantly, relevant organ dysfunction as a key element of definition was present in all patients, indicated by the Sequential Organ-Failure Assessment score (SOFA) (Table 1).

**Table 1:** Baseline characteristics of patients and healthy volunteers.

	**Cohort 1**			**Cohort 2**			**Cohort 3**
	**Sepsis**	**Post-OP**	**Healthy**	**Sepsis**	**Post-OP**	**Healthy**	**Trauma**
n =	50	20	20	30	30	30	36
Age (years)	66 (41–88)	65 (49–80)	30 (23–44)	60 (20–84)	64 (36–85)	23 (19–45)	49 (18–85)
Male sex	38 (76)	10 (50)	5 (25)	21 (70)	23 (77)	12 (40)	27 (75)
BMI (kg/m2)	27.2 (18.8–45.9)	24.9 (29.2–37.2)	–	24.8 (18.2–47.8)	27.4 (16.6–41.2)	23.0 (18.0–28.4)	25.4 (19.5–38.6)
**CLINICAL SCORES**							
SOFA	11 (6–18)	–	–	14 (6–20)	–	–	6 (1–15)
ISS	–	–	–	–	–	–	24 (17–34)
**SITE OF INFECTION (MULTIPLE NAMING POSSIBLE)**							
Surgical site	30 (60)	–	–	12 (40)	–	–	–
Abdominal	45 (90)	–	–	11 (36.7)	–	–	–
Urinary tract	1 (2)	–	–	1 (3.3)	–	–	–
Lung	10 (20)	–	–	5 (16.7)	–	–	–
Other	1 (2)	–	–	6 (20)	–	–	–
**LABORATORY VALUES**							
Leucocytes (× 10^3^/μl)	12.1 (1.7–76.0)	6.9 (4.8–11.2)	–	19.2 (1.2–52.6)	12.2 (4.4–32.8)	6.6 (4.1–11.4)	8.9 (4.5–19.2)
CRP (mg/l)	190.7 (19.2–430.3)	5.5 (2.0–92)	–	218.9 (49.6–522.6)	2.7 (2.0–74.2)	2 (2.0–5.0)	16.7 (0.0–161.0)
PCT (μg/l)	8.3 (0.1–288.5)	–	–	17.5 (0.3–185.9)	0.1 (0.05–1.5)	0.05 (0.05–0.05)	0.7 (0.05–13)
**OUTCOME**							
28-day-mortality	11 (22)	0 (0)	0 (0)	12 (40)	2 (7)	0 (0)	1 (3)

*Data are presented either as median (min–max) or as number (percentage) in case of “Male sex” and “28-day-mortality.” BMI, Body mass index; APACHE II, Acute physiology and chronic health evaluation score II; SAPS II, Simplified acute physiology score II; SOFA, Sequential organ failure assessment score; ISS, Injury severity score; Post-OP, postoperative*.

Cohort 3 (SPRINT; German Clinical Trials Register ID: DRKS00010991; Ethical committee of the Medical Faculty of the Justus-Liebig-University Giessen: 164/14) contains samples of 36 adult patients (as available from originally 50 patients) with severe traumatization (Injury Severity Score (ISS) ≥ 16), drawn on admission (initial), 24, 48, 72, and 96 h later. Patients with known chronic viral infections have been excluded (Koch et al., 2018).

To rule out an influence of age and sex on sDLL1 plasma concentrations, 90 anonymized healthy control samples of both sex and different age groups (18–27, 28–37, 38–47, 48–57, and 58–67 years, *n* = 9 each group and sex) were obtained from the Blood Donor Biobank (Bavarian Red Cross Blood Donor Service) and analyzed.

### Measurement of DLL1

Quantification of soluble DLL1 in plasma has been performed using a commercially available ELISA kit (RayBiotech Life, Inc., Norcross, USA) according to the manufacturer's instructions. Importantly, all samples were diluted 1:30 (or higher if demanded by the concentration) with the supplied Assay Diluent A before quantification to lie within the calibration curve and to minimize interfering matrix effects. Absorbance measurements have been performed on an ELx808 microplate reader (BioTek Instruments, Inc., Winooski, USA) with a subsequent automatized calculation of concentrations within the corresponding Gen5 software (BioTek Instruments, Inc., Winooski, USA).

Measurements of leucocytes, CRP and PCT have been performed in the routine laboratories of each study site.

### Statistical Analysis

All visualizations and statistical analysis have been conducted with GraphPad Prism (version 8.1.2, GraphPad Software, Inc., La Jolla, USA). All CRP and PCT values below internal reference range (2 mg/l and 0.05 μg/l) were set to 2 and 0.05 μg/l, respectively. Scatter plots containing single data points are used for visualization, with medians indicated within. If not stated otherwise, numbers reported are median values (95% confidence interval).

Group comparisons between corresponding timepoints of postoperative and sepsis patients within cohort 1 and 2 were conducted using Kruskal-Wallis test, followed by pairwise comparison with Dunn's post-test (corrected for multiple testing). Healthy controls were compared to timepoints 0, 24, and 48 h of patients with sepsis. Influence of age and sex was assessed by ordinary two-way-ANOVA in the dataset of healthy donors.

To assess the diagnostic performance of DLL1 in comparison to leucocytes, CRP and PCT, a pooled *Area Under Receiver Operator Characteristic* (AUROC) analysis was performed: (1) all samples from patients with sepsis and after surgery (0, 24, and 48 h) as well as from patients with trauma (all time points) and healthy volunteers were selected, from which measurements of all four biomarkers were available. (2) Samples were grouped into “Sepsis” (*n* = 148) or “Control” (*n* = 201; healthy volunteers, surgical and trauma patients). Area under curve (AUC) and the 95% confidence interval were reported as global indicators of discriminatory performance. Same approach was repeated to evaluate the prognostic value of DLL1 in regard to 28-day mortality within the patients with sepsis. *Post-hoc* power calculation was performed using G^*^Power (version 3.1.9.3., free from University of Düsseldorf) (Faul et al., 2007).

To identify the cut-off value corresponding to the best combination of sensitivity and specificity, Youden index was calculated and the maximum value selected. Subsequently, the accuracy of each marker was calculated by applying the identified cut-off to the full ROC analysis cohort (*n* = 349). Furthermore, the correlation between markers was assessed by Spearman-Rho test.

## Results

### The Study Cohorts

In total, 80 patients with sepsis were available for this secondary analysis. In both cohorts 1 and 2, the majority of these patients were male and suffered from abdominal, respectively, surgical site-associated infection as the source of sepsis (Table 1), with a total of 15 patients (18.6%) also having evidence for a (co-)infection of the lung. Both cohorts were similar regarding disease severity as depicted by SOFA score. However, mortality was higher in Cohort 2 (C2, 40%) compared to Cohort 1 (C1, 22%).

Groups of patients with sepsis and patients after surgery (*n* = 50) were comparable in age, while healthy volunteers (*n* = 50), and trauma patients (*n* = 36) were substantially younger. CRP, leucocytes, and PCT were partly available from post-surgery patients and healthy volunteers, while biomarkers were consistently available for patients with sepsis (Supplementary Figure 1). Most importantly, surgical patients exhibited a delayed increase of CRP, PCT, and leucocytes (Supplementary Figures 1A–F), while trauma patients primarily increased CRP, as well as PCT plasma levels (Supplementary Figures 1G–I). That kinetics are indicative of a sterile systemic inflammatory response syndrome (SIRS), blurring the biomarker-based diagnosis of emerging infections.

### The Kinetics of DLL1 in Sepsis and Trauma

**Figure 1:**
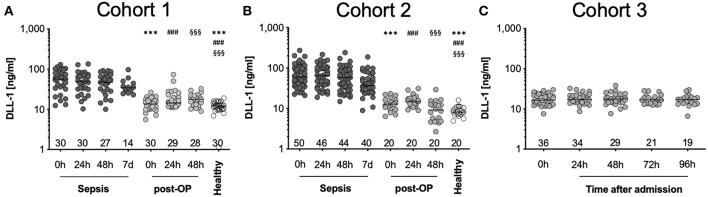
Distribution of DLL1 plasma concentrations in the study cohorts. **(A,B)** depicts measurements of sepsis studies and **(C)** of trauma patients. Bottom numbers indicate available samples on each timepoint. Horizontal line depicts median, y-axis has been set to log-scale. ^***^/###/§§§ *p* < 0.001 vs. “Sepsis 0 h,” “Sepsis 24 h,” or “Sepsis 48 h,” respectively. Post-OP, postoperative.

We evaluated the kinetics of DLL1 in different groups of patients as well as healthy volunteers. Latter exhibited median plasma concentrations 12.1 ng/ml (CI: 10.6–13.2 ng/ml; C1) and 8.2 ng/ml (95%CI: 7.7–10.1 ng/ml; C2) (Figures 1A,B). We found a strong increase in plasma concentrations already at the onset of sepsis [0 h, 56.5 ng/ml (C1), 60.5 ng/ml (C2)] (Figures 1A,B, Supplementary Table 1). DLL1 concentrations remained elevated after 24 h (48.6 and 64.7 ng/ml), 48 h (48.2 and 58.1 ng/ml), and even 7 days later (34.3 and 37.4 ng/ml), compared to healthy reference values (Figures 1A,B, Supplementary Figure 2, Supplementary Table 1). To examine the impact of sterile inflammation on DLL1 concentrations, we measured two stress test cohorts: patients after surgery and patients with severe multiple injuries, both groups involving extensive tissue damage and subsequent sterile inflammation as described above. In patients after surgery, DLL1 increased slightly from 13.6 to 17.4 ng/ml after 48 h (in C1) and from 12.8 to 15.2 ng/ml after 24 h (in C2) (Figures 1A,B, Supplementary Table 1). In line, patients with multiple injuries presented—compared to healthy volunteers—slightly increased plasma concentrations from admission (0 h, 16.4 ng/ml; 16.2–20.1 ng/ml) until day 4 after trauma (17.0 ng/ml; 14.5–19.7 ng/ml) (Figure 1C, Supplementary Table 1). Importantly, after analysis of the healthy donor cohort, age and sex did not exert an effect on sDLL1 concentrations, neither individually, nor additive (Supplementary Figure 3).

In summary, DLL1 is increased exclusively in sepsis, while sterile insults as major surgery and severe trauma do not influence its generation.

### DLL1 Has Superior Diagnostic Performance

**Figure 2:**
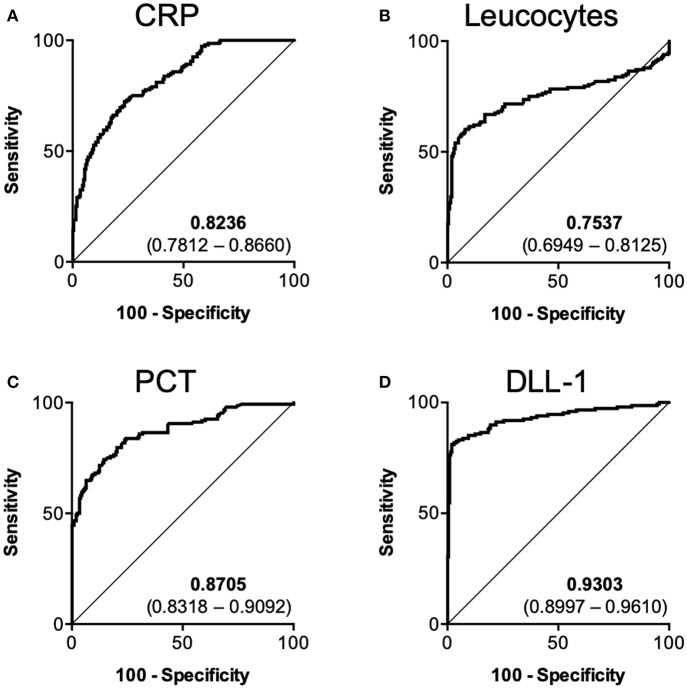
Diagnostic performance of DLL1 in comparison to established biomarkers. Pooled ROC analysis containing 148 sepsis samples vs. 201 control samples. **(A)** CRP (C-reactive protein), **(B)** Leucocytes, **(C)** PCT (Procalcitonin), **(D)** DLL1 (Delta-like 1). Numbers within subpanels indicate area under curve (lower–upper 95% confidence interval).

Next, we investigated the value of DLL1 as a diagnostic marker for the differentiation between sterile inflammation and sepsis. Therefore, we pooled all samples from which leucocytes, CRP and PCT data were available and grouped them into “sepsis” (*n* = 148) and “controls” (*n* = 201). An correlation analysis of all patients with sepsis (0, 24, and 48 h) yields no correlation of DLL1 with leucocytes (−0.03411 (CI: −0.1674–0.1004; *p* < 0.00001; *n* = 227), and only weakly with CRP (0.2253 (CI: 0.0938–0.3490; *p* < 0.00001; *n* = 226), as well as with PCT (0.3655 (CI: 0.2140–0.4998; *p* < 0.00001; *n* = 151). Next, AUROC analysis was conducted and revealed AUCs of 0.8236 (0.7812–0.8660) for CRP (Figure 2A), 0.7573 (0.6949–0.8125) for leucocytes (Figure 2B), 0.8705 (0.8318–0.9092) for PCT (Figure 2C), and 0.9303 (95%CI: 0.8997–0.9610) for DLL1 (Figure 2D). *Post-hoc* analysis revealed a power of 1.0 for the discriminatory value of DLL1. The best cut-off values (and the corresponding combination of sensitivity/specificity) of the individual markers were extracted by maximum Youden index procedure: 159.4 mg/l CRP (72.3% sensitivity/76.6% specificity), 15.2 μl leucocytes (60.1% sensitivity/92.0% specificity), 2.5 μg/l PCT (74.3% sensitivity/85.6% specificity), and 29.7 ng/ml DLL1 (81.8 sensitivity/97.0 specificity). Finally, the accuracy of each marker was calculated by applying the given thresholds to the full cohort, yielding a superior accuracy of 91% for DLL1, compared to 75, 79, and 81% for CRP, leucocytes, and PCT, respectively. Regarding outcome prediction (28-day survival), plasma DLL1 failed to be of prognostic value (Supplementary Figure 4). However, statistical power of these analysis is low, with 0.0641 (Onset) and 0.195 (24 h), needing replication in larger cohorts.

## Discussion

In line with our prior experimental finding of DLL1 induction upon monocyte activation (Hildebrand et al., 2018), we found plasma levels of DLL1 to be strongly elevated in septic patients. In contrast, neither surgical trauma nor severe injuries lead to an increase of it. As these conditions are well-known to induce sterile inflammatory responses of the body, DLL1 represents a robust and specific biomarker for sepsis. Compared to routine laboratory markers, especially to PCT, DLL1 exhibits a superior accuracy for diagnosis, but not for prognosis, of bacterial infection.

The syndrome sepsis can result from various infectious encounters of patients already embedded in complex clinical contexts, fueling an interwoven machinery of disturbed response systems of the host (Angus and van der Poll, 2013). Importantly, phenotypically similar host responses can be mounted upon, e.g., surgery (Dabrowska and Słotwinski, 2014), severe injury (Lord et al., 2014), and ischemic stroke (Courties et al., 2014), hampering the performance of existing biomarkers to discriminate an infection-related host response to sterile responses. The most prominent biomarker PCT, has been approached in a plethora of studies and despite the availability of large, comparative datasets, its real-life utility is controversially debated since many years (Simon et al., 2004; Tang et al., 2007; Wacker et al., 2013; Wu et al., 2017). Only recently, a study including patients presenting with SIRS on ICU revealed that no humoral biomarker currently available is capable to stratify patients with critical illness of non-infectious and infectious origin (Parlato et al., 2018). Among all markers examined, CRP levels and HLA-DR expression showed the best diagnostic performance, but both still exhibit values too low for routine use.

The intriguing difference of sDLL1 compared to CRP and PCT is its persistence over time. While established marker substantially respond to intensive care therapy, DLL1 remains elevated for 7 days. It is unclear, if this phenomenon is due to steady formation of new sDLL1 or if the half-life is just substantially longer. In any case, DLL1 remains informative for diagnosis over a sustained period of time, thereby limiting its use for therapy guidance. Many markers used in clinical routine exert redundant informational content. As we are able to show its weak correlation to other markers, the use of sDLL1 might add further value to diagnosis.

While the cellular origin of the high levels of sDLL1 observed in our patients with sepsis remains speculative, the mechanism of formation is known: transmembrane DLL1 is cleaved by a combined ADAM protease and γ-secretase activity upon binding to Notch receptor and therefore, sDLL1 is a surrogate of recent Notch signaling (Six et al., 2003; Dyczynska et al., 2007). Biological processes of utmost importance in host defense are monocyte activation and maturation in the circulation. Activation of the DLL1-Notch axis by endothelial cells has been shown to be a crucial trigger for the conversion of classical into alternative (non-classical) monocytes under steady state (Gamrekelashvili et al., 2016), as well as for the maturation of tissue macrophage upon ischemia toward a pro-resolving and repair phenotype (Krishnasamy et al., 2017). The resulting non-classical monocytes exhibit an impaired antigen-presentation capacity, but increased expression of the immune checkpoint *Programmed Death*-1 (PD-1) and its ligand PD-L1 (Ferreira da Mota et al., 2018). This is in line with our finding of an inter-monocyte activation of the Notch axis, resulting in a comparable phenotype (Hildebrand et al., 2018). Taken together, beside monocytes one might speculate the endothelium as a relevant source of cleaved DLL1 observed in plasma of patients with sepsis as a consequence of signaling. Considering the cytokine-mediated capillary leakage syndrome as a well-known clinical hallmark of sepsis (Siddall et al., 2017), the Notch axis might be a critical molecular hub in the pathophysiology, warranting further research.

A series of publications evaluated the use of sDLL1 as a biomarker in different diseases. In a cohort of 136 patients with symptomatic aortic stenosis, Abraityte and colleagues found a prognostic value of sDLL1, with low and high plasma levels indicating poor outcome (Abraityte et al., 2015). However, “high” plasma levels were already defined as ≥6.93 ng/ml, which is substantially below the threshold we proposed for the diagnosis of infection and lies well within the range of the healthy volunteers we discovered.

The same group also showed elevated levels in a cohort of patients with chronic heart failure compared to healthy volunteers (Norum et al., 2016). Again, sDLL1 negatively correlated with diastolic function, exercise capacity, as well as outcome. The reported concentrations were well below the values we observed in our cohorts, with 7.4 ng/ml indicated as upper tertile of the cohort. Based on these findings, Norum et al. (2017) further examined plasma of patients suffering from dilated cardiomyopathy, as these commonly present with diastolic dysfunction. Importantly, in contrast to the initial publications, levels of sDLL1 in healthy controls were identical to the values we observed, with patients of high disease severity presenting slightly elevated levels (approximately median 12–13 ng/ml, as results are not given in publication). Furthermore, in both patient groups with dilated cardiomyopathy and chronic heart failure, sDLL1 correlated well with markers of impaired kidney function (e.g., creatinine). Patients with sepsis often impose with renal dysfunction as a consequence of inflammation-induced acute kidney injury, potentially leading to an accumulation of sDLL1 (Zarbock et al., 2014). Further studies need to unravel the association between DLL1 and kidney function during sepsis. Also, looking in urine might be of interest to evaluate its use as biomarker of kidney function.

Besides heart diseases, also patients with non-small cell lung cancer (NSCLC) and chronic obstructive pulmonary disease (COPD) were recently assessed for sDLL1 (Berg et al., 2018). Plasma levels of DLL1 differed between the two cohorts, ranging from 3.8 to 22.5 ng/ml in NSCLC and from 8.1 to 27.8 ng/ml in COPD patients. Apart from internal medicine, patients with schizophrenia, as well as bipolar disorders were shown to possess elevated levels of sDLL1 compared to healthy controls (Hoseth et al., 2018). Conflicting, this study reports substantially lower levels of sDLL1 (median 4.5 ng/ml) in healthy volunteers, as compared to their own earlier studies and to our results. Two challenges might be underlying this phenomenon: first, there is no standardized assay for the quantification of DLL1 and secondly, no indication is given how the assay was performed in terms of sample dilution. As plasma and serum are complex matrixes, interferences cannot be excluded, potentially introducing a technical bias.

In a combined study on two cohorts of patients after heart transplantation, sDLL1 concentrations have been found to be elevated compared to healthy controls (Norum et al., 2019). Moreover, time since transplantation, as well as the immunosuppressive medication used (everolimus or calcineurin inhibitor) were shown to influence sDLL1 concentrations. The authors reported median values between 12.8 and 26.6 ng/ml in different subgroups. If looking in detail, the highest values were reported in patients with acute rejections on baseline (median: 26.6 ng/ml; IQR: 23.1–31.0 ng/ml). These values are ranging into the threshold proposed from us for diagnosis of infection and therefore might represent a limitation of our marker. Importantly, in their publication Norum and colleagues also identified expression of DLL1 on T cells, endothelial cells, and vascular smooth muscle cells and its release upon activation with cytokines. This further substantiates the hypothesis of endothelial cells as relevant source of circulating sDLL1.

Results of one earlier study hinted, comparable to ours, toward a diagnostic value of DLL1 for infection: By assessing sDLL1 levels in cerebrospinal fluid of patients with HIV with suspected *Mycobacterium tuberculosis* infection, the researchers found a cut-off value of 1.15 ng/ml associated with an excellent specificity of 98%, but a low sensitivity of only 32% (Bahr et al., 2018). However, this clinical context is very special and CSF is not as easily obtainable as blood. Nevertheless, one important conclusion can be drawn from this study, despite in need of clarification in the blood: chronic HIV infection does not seem to elevate DLL1 levels *per se*, but only when bacterial infection occurs in addition.

Our study implies several limitations, not lastly due to its characteristic as a secondary analysis of three independent cohorts. No matching for sex and age was performed in the primary studies, leading to skewed demographic compositions. Using a separate dataset of healthy donors, we are able to prove no clear influence of either age or sex on sDLL1 concentrations. However, larger cohorts are necessary to define definite reference values. From a technical perspective, to prove DLL1's potential as a biomarker of bacterial infection (and other pathologic conditions), several aspects need to be ensured: First, a standardized assay needs to be developed and used for quantification, enabling definite cut-off determination. Secondly, sample pre-analytic must be harmonized with respect to anticoagulation of drawn blood, processing, and storage. Especially analytical stability is a crucial aspect of biomarker assessment und clinical usability. It must be carefully revisited for DLL1, as our samples have been stored for an extended time frame before analysis and we cannot rule out degradation of sDLL1. However, as all cohorts recruited patients and controls during the same period, bias within the cohorts is limited. Also, considering the high amounts of sDLL1 consistently present in patients with sepsis, the value of DLL1 as a biomarker for the identification of bacterial infection in critically ill patients remains unchallenged, with the potential exception of patients after heart transplantation. This limit needs to be verified in further studies, including also patients after transplantation of other solid organs. Importantly, the real-life approach of Parlato and colleagues should serve as a role model for the design of future biomarker studies, evaluating the marker of interest directly within the setting of interest.

In conclusion, by combining several cohorts of patients, we identified plasmatic sDLL1 to be a potential new host-derived biomarker with high diagnostic accuracy for sepsis. Its superior sensitivity and specificity compared to CRP and PCT must be confirmed in independent cohorts. As it results from a specific process inherent to the syndromes' pathophysiology, the DLL1-Notch axis might serve as a potential theranostic target in sepsis as well.

## Data Availability

The raw data supporting the conclusions of this manuscript will be made available by the authors, without undue reservation, to any qualified researcher.

## Ethics Statement

The study protocol of each study contained was assessed and positively evaluated by the local ethics committee (S-247/2014 and S-097/2013, Ethical Committee I of the Medical Faculty Heidelberg; 164/14, Ethical Committee of the Medical Faculty of the Justus-Liebig-University Giessen). Furthermore, the studies were registered in the German Clinical Trials Register (IDs: DRKS00008090, DRKS00005463, DRKS00010991). Informed consent was obtained from the patients or, if not possible due to sedation or mental deterioration, from the legal representative.

## Author Contributions

DH contributed to the study concept, conducted sample measurements, interpreted the results, and drafted and wrote the manuscript. SD, CK, and FS came up with the primary study concept, recruited patients, and interpreted results. SR and ES recruited patients, interpreted results, and drafted the manuscript. MS contributed to the primary study concept, recruited patients, interpreted results, and drafted the manuscript. KH and MW contributed to the study concept, interpreted results, and drafted the manuscript. TB contributed to the primary and secondary study concepts, interpreted results, and drafted the manuscript. FU contributed to the study concept, conducted sample measurements, data analysis, and drafted and wrote the manuscript.

### Conflict of Interest Statement

DH, KH, MW, and FU hold the worldwide intellectual property rights for the use of DLL1 as diagnostic marker for severe infections (PCT/EP2018/079273 and EP17198330). The remaining authors declare that the research was conducted in the absence of any commercial or financial relationships that could be construed as a potential conflict of interest.
