# Supplementary material for: Host-Derived Delta-Like Canonical Notch Ligand 1 as a Novel Diagnostic Biomarker for Bacterial Sepsis—Results From a Combinational Secondary Analysis
Source: Front Cell Infect Microbiol. 2019 Jul 23;9:267. doi: 10.3389/fcimb.2019.00267 (PMC6663974; doi:10.3389/fcimb.2019.00267)
Supplement: Supplementary file 1 [file Table_1.pdf]

Supplementary table 1: Plasma DLL-1 concentrations in patients and volunteers of studied cohorts over time. All values are given in ng/ml. CI: 95% confidence interval. Post-OP: postoperative.

**Cohort 1**								
	Sepsis				Post-OP			Healthy
Timepoint	0h	24h	48h	7d	0h	24h	48h	
n=	30	30	27	14	30	29	28	30
Median	56.52	48.69	47.19	34.32	13.62	14.25	17.38	12.12
CI low	47.82	41.82	39.77	28.57	12.39	14.54	15.50	10.65
CI up	72.77	64.23	59.21	50.93	16.00	24.57	19.94	13.21
**Cohort 2**								
	Sepsis				Post-OP			Healthy
Timepoint	0h	24h	48h	7d	0h	24h	48h	
n=	50	46	44	40	20	20	20	20
Median	60.54	64.73	58.11	37.38	12.83	15.21	9.21	8.22
CI low	62.60	61.46	51.60	36.73	11.28	13.41	7.18	7.67
CI up	92.23	88.03	78.93	58.35	15.82	18.96	13.00	10.06
**Cohort 3**								
	Trauma							
Timepoint	0h	24h	48h	72h	96h			
n=	36	34	29	21	19			
Median	16.44	17.15	17.61	16.72	17.01			
CI low	16.23	16.64	15.87	15.52	14.48			
CI up	20.07	20.27	20.60	20.11	19.71			
