# Supplementary material for: Host-Derived Delta-Like Canonical Notch Ligand 1 as a Novel Diagnostic Biomarker for Bacterial Sepsis—Results From a Combinational Secondary Analysis
Source: Front Cell Infect Microbiol. 2019 Jul 23;9:267. doi: 10.3389/fcimb.2019.00267 (PMC6663974; doi:10.3389/fcimb.2019.00267)
Supplement: Supplementary file 2 [file Image_1.pdf]

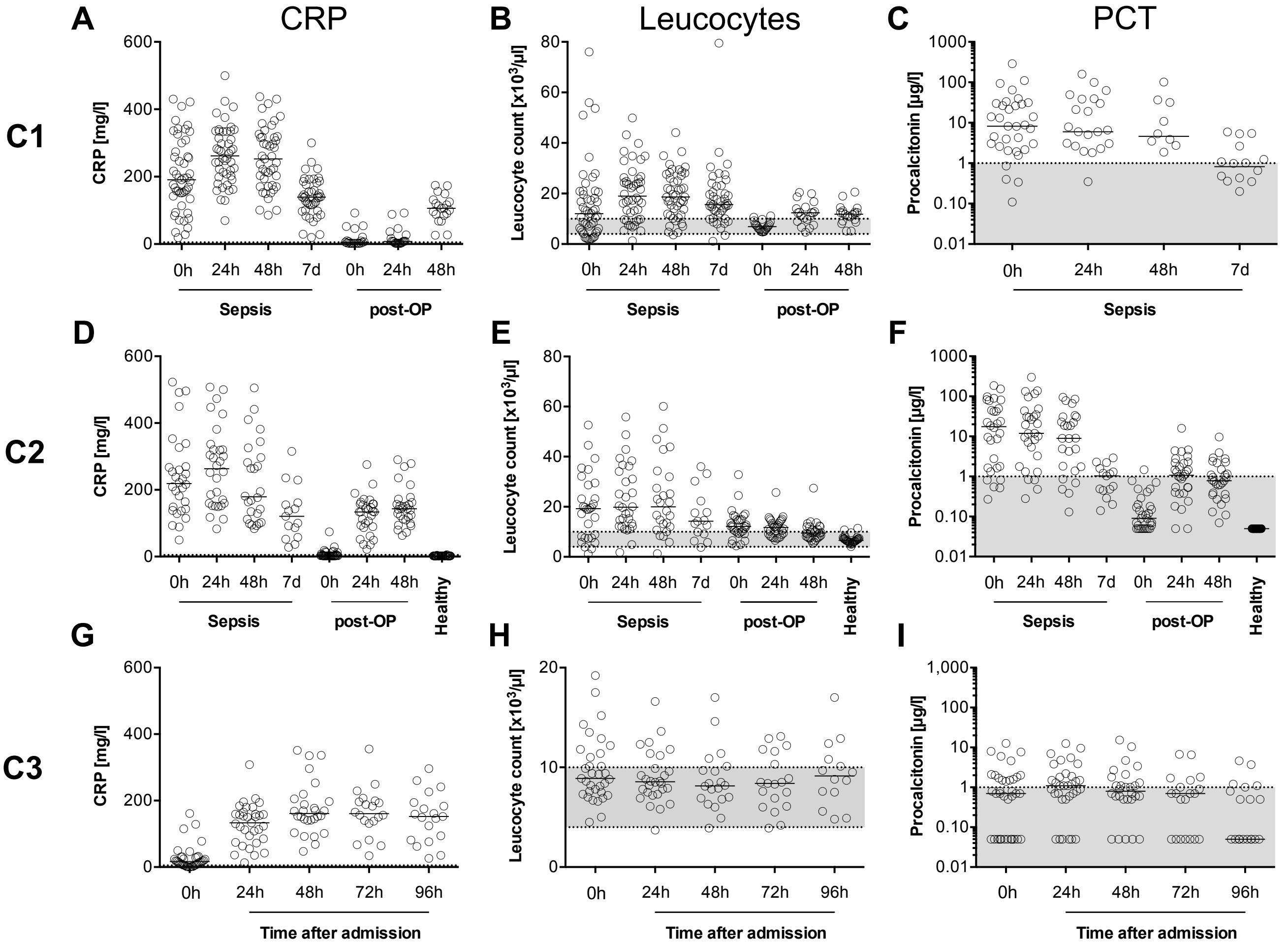

Supplementary figure 1: Distribution of routine biomarker values in the study cohorts. (**A**) - (**C**) Cohort 1, (**D**) - (**F**) Cohort 2, (**G**) - (**I**) Cohort 3. For each biomarker, the reference range was incorporated into the figures as grey fill between dashed line: <5mg/l for CRP (**A**, **D**, **G**), 4 – 10x10^3 leucocytes/µl (**B**, **E**, **H**), <1µg/l for PCT (**C**, **F**, **I**). C1-3: Cohort 1-3.
