# Supplementary material for: Host-Derived Delta-Like Canonical Notch Ligand 1 as a Novel Diagnostic Biomarker for Bacterial Sepsis—Results From a Combinational Secondary Analysis
Source: Front Cell Infect Microbiol. 2019 Jul 23;9:267. doi: 10.3389/fcimb.2019.00267 (PMC6663974; doi:10.3389/fcimb.2019.00267)
Supplement: Supplementary file 3 [file Image_2.pdf]

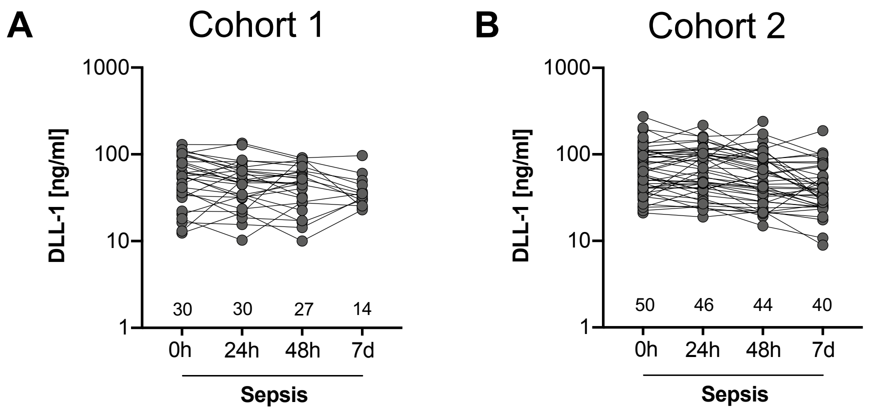

Supplementary figure 2: Longitudinal development of DLL1 plasma concentrations in patients with sepsis. Bottom numbers indicate available samples on each timepoint.
