# Supplementary material for: Host-Derived Delta-Like Canonical Notch Ligand 1 as a Novel Diagnostic Biomarker for Bacterial Sepsis—Results From a Combinational Secondary Analysis
Source: Front Cell Infect Microbiol. 2019 Jul 23;9:267. doi: 10.3389/fcimb.2019.00267 (PMC6663974; doi:10.3389/fcimb.2019.00267)
Supplement: Supplementary file 4 [file Image_3.pdf]

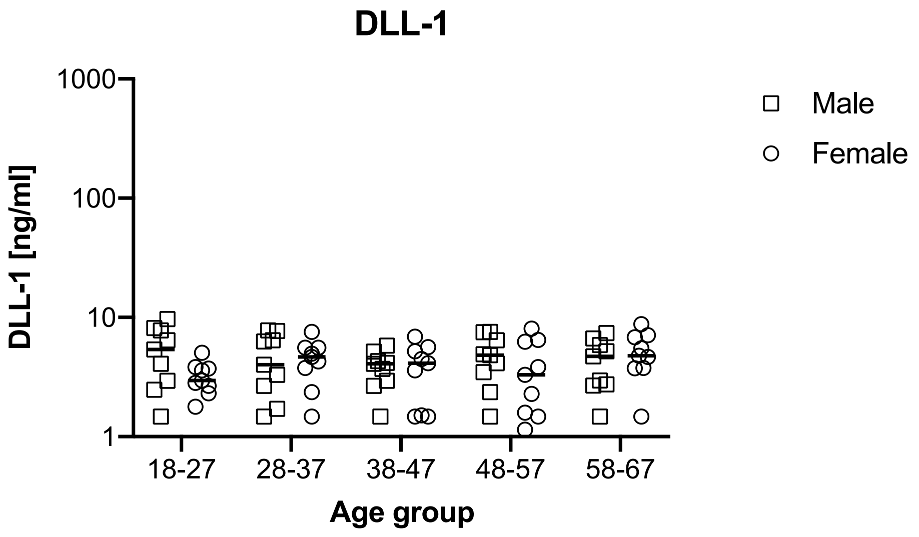

Supplementary figure 3: Distribution of sDLL1 plasma concentrations within the healthy donor cohort, grouped for age groups and sex.
