# Supplementary material for: Host-Derived Delta-Like Canonical Notch Ligand 1 as a Novel Diagnostic Biomarker for Bacterial Sepsis—Results From a Combinational Secondary Analysis
Source: Front Cell Infect Microbiol. 2019 Jul 23;9:267. doi: 10.3389/fcimb.2019.00267 (PMC6663974; doi:10.3389/fcimb.2019.00267)
Supplement: Supplementary file 5 [file Image_4.pdf]

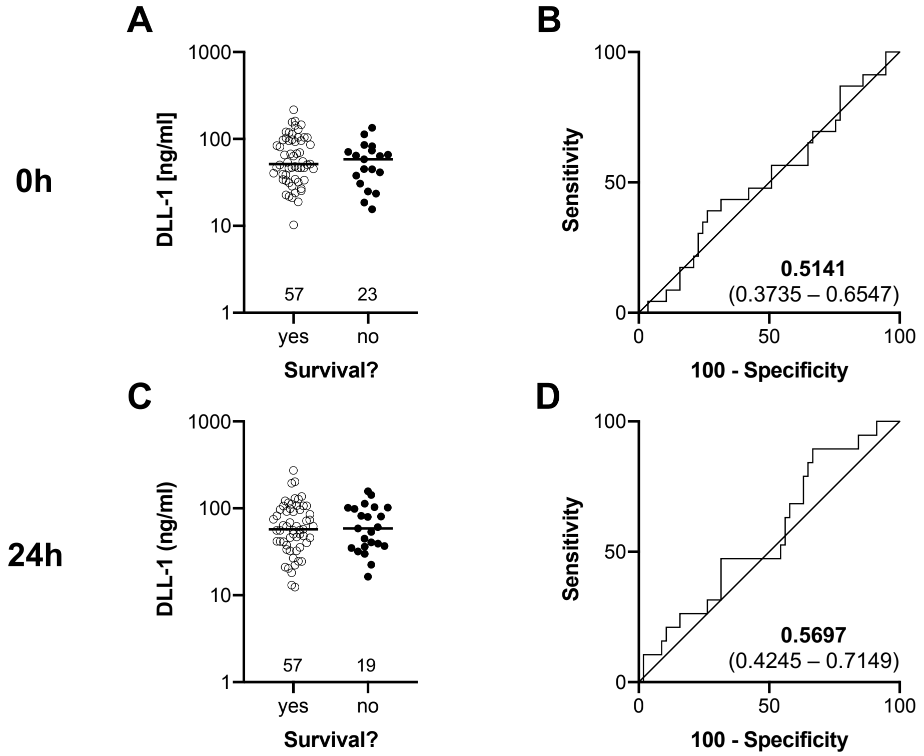

Supplementary figure 4: Prognostic value of plasma DLL1. Plasma concentrations of DLL1 at Onset (**A**) or 24h (**C**), grouped for 28-day survival. Horizontal line represents median, numbers indicate patients in analysis. Results of AUROC analysis at Onset (**B**) or 24h (**D**) for 28-day survival. Numbers indicate AUC (with 95% confidence interval).
